# Antibiogram and virulence profiling reveals multidrug resistant *Staphylococcus aureus* as the predominant aetiology of subclinical mastitis in riverine buffaloes

**DOI:** 10.1002/vms3.942

**Published:** 2022-09-22

**Authors:** M. Nazmul Hoque, Anup Kumar Talukder, Otun Saha, Mehedi Mahmudul Hasan, Munawar Sultana, ANM Aminoor Rahman, Ziban Chandra Das

**Affiliations:** ^1^ Department of Gynecology Obstetrics and Reproductive Health Bangabandhu Sheikh Mujibur Rahman Agricultural University (BSMRAU) Gazipur Bangladesh; ^2^ Department of Microbiology Noakhali Science and Technology University Noakhali Bangladesh; ^3^ Department of Microbiology University of Dhaka Dhaka Bangladesh; ^4^ Department of Fisheries and Marine Science Noakhali Science and Technology University Noakhali Bangladesh

**Keywords:** antibiotic sensitivity, buffalo, virulence, enterotoxins, subclinical mastitis, *Staphylococcus aureus*

## Abstract

**Background:**

*Staphylococcus* spp. are the major causal agents of mastitis in dairy animals worldwide leading to profound economic losses and public health threats. Recently, *Staphylococcus aureus* has emerged as a multidrug resistant and zoonotic pathogen. This study aimed to characterize *S. aureus* in subclinical mastitis (SCM) milk samples of riverine buffaloes in Bangladesh through antibiogram and virulence gene(s) profiling, and 16S rRNA gene sequencing.

**Method:**

We characterized *S. aureus* in SCM milk samples (N = 500) of riverine buffaloes through antibiogram and virulence gene(s) profiling, and 16S rRNA gene sequencing.

**Results:**

Out of 500 milk samples tested, 188 (37.6%) were found positive for SCM. From 188 SCM samples, 291 isolates were obtained with a prevalence of *S. aureus* in 37.4% (109/291) isolates. Phylogenetic analysis revealed the evolutionary divergence of *S. aureus* isolates in bubaline SCM milk samples. The antibiogram profiling showed that about 96.0% *S. aureus* isolates were multidrug resistant (MDR). Notably, 29 and 16 isolates harboured methicillin‐resistant (*mecA*) and panton‐valentine leucocidin (*pvl*) genes, respectively, and 46 plasmid‐bearing isolates were MDR. Nine *Staphylococcal* enterotoxins (SEs/SEls) including *sea* (11.9%), *sec* (7.4%), *sed* (4.6%), *seg* (3.7%), and *seh* (3.7%) were detected with 72.48% toxinotypes comprising a single gene.

**Conclusion:**

This study therefore suggests *S. aureus* as the single‐most aetiology (∼37.0%) of SCM in riverine buffaloes, and emergence of MDR, enterotoxin producing, and virulent *S. aureus* strains could impose potential threats to animal welfare and public health.

## Graphical abstract

Graphical abstract showing overview of the subclinical mastitis (SCM) study in buffalo cows. This study aimed to characterized *S. aureus* in SCM milk samples of riverine buffaloes through antibiogram and virulence gene(s) profiling, and 16S rRNA gene sequencing. Overall, phylogenetically diverse, multidrug resistant (MDR), virulent, enterotoxin producing *S. aureus* strains were found as the predominant aetiology of SCM in riverine buffalo cows. This study therefore suggested *S. aureus* as the single‐most aetiology (∼37.0%) of SCM in riverine buffaloes, and emergence of MDR, enterotoxin producing, and virulent *S. aureus* strains could impose potential threats to animal welfare and public health.

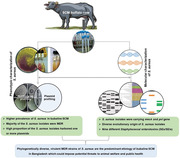

## 1 INTRODUCTION

Mastitis is one of the most prevalent, costly and complex diseases in the dairy industry with economic losses attributable to reduced milk production, discarded milk, early culling, veterinary services, and labour costs (Hoque et al., 2018; Sharma et al., 2012; Thompson‐Crispi et al., 2014). The disease is thought to be one of the most important reasons for reduced milk production, shortage in per capita milk consumption, and economic losses in Bangladesh. The milk of lactating cows serves as the best media for the proliferation of various pathogenic, opportunistic, and spoilage microorganisms (Hoque et al., 2019; Zayda et al., 2020) and can influence the pathophysiology of mastitis (Hoque, Istiaq, Rahman, et al., 2020). Depending on the host–pathogens interactions, mastitis can manifest in clinical mastitis (CM) or subclinical mastitis (SCM) form (Hoque, Istiaq, Clement, et al., 2020), of which SCM is the most common form in all dairy animals and responsible for greater economic losses (Hoque et al., 2015; Salvador et al., 2012). In dairy cows, SCM is 15–50 times more prevalent than CM, is of longer duration and difficult to detect (Sarker et al., 2013) which attracts prompt attention to its early diagnosis in the dairy herds (Hoque et al., 2018, 2015). Almost every dairy herd has cows with SCM, and a variety of pathogens can establish chronic infections which may only occasionally manifest the clinical signs of mastitis (Hoque et al., 2018, 2015).

*Staphylococcus aureus* is a ubiquitous facultative zoonotic pathogen and can cause diseases in both humans and domestic animals (El‐Ashker et al., 2015; Hoque et al., 2018; Shrestha et al., 2021). Methicillin‐resistant *S. aureus* (MRSA) strains known as “resistant staph” or “superbug” are a major cause of nosocomial infections that lead to increased mortality rates and a substantial increase in human health care costs (Shrestha et al., 2021). *Staphylococcus aureus* is an increasingly recognized etiologic agent of bovine (Hoque et al., 2018; Rossi et al., 2019) and bubaline (de Medeiros et al., 2011; Guccione et al., 2020) mastitis and has been remaining as the most frequently isolated bacterium from udder infections especially in herds with high prevalence of SCM. Furthermore, *S. aureus* is categorized as one of the major pathogens causing mastitis in dairy animals (El‐Ashker et al., 2015) and responsible for about one‐third of cases of the SCM (Hoque et al., 2018; Haran et al., 2012; Shome et al., 2011). The ability of *S. aureus* to cause mastitis is probably due to the expression of various toxins, virulence factors, and cell wall adhesion proteins (El‐Ashker et al., 2015; Haran et al., 2012). The bacterium has the ability to survive phagocytosis in the udder and often causes chronic inflammation (El‐Ashker et al., 2015; Hoque et al., 2018). Therefore, early diagnosis of SCM is essential because changes in udder tissue take place much earlier than they become apparent.

The resistance of *S. aureus* to commonly used antibacterial agents presents an increasing challenge and can complicate the treatment of mastitis. High prevalence of antimicrobial resistance (AMR) in both animal and human *S. aureus* isolates could be due to widespread, uncontrolled, and indiscriminate use of antibiotics (Al Amin et al., 2020, 2022). The use of broad‐spectrum antibiotics creates a selective pressure on the bacterial flora, thus increasing the emergence of new antibiotic‐resistant bacteria (Bantawa et al., 2019; Hoque, Istiaq, Clement, et al., 2020). Although the prevalence of MRSA strains in mastitis seems to be generally low in buffalo farms (El‐Ashker et al., 2015), the emergence of antibiotic‐resistant microorganisms poses a potential public health hazard. More recently, multidrug resistance (MDR) was detected in both coagulase‐positive and coagulase‐negative *Staphylococci* isolated from buffaloes and cows mastitis (Aires‐de‐Sousa et al., 2007; Dorgham et al., 2013). Furthermore, the treatment efficacy of *S. aureus* mastitis is usually disappointing because of excessive damages in the mammary tissues and drugs are not able to penetrate to all infected sites (Bantawa et al., 2019; Hoque, Istiaq, Clement, et al., 2020). This bacterium also suppresses phagocytosis and cell‐mediated immunity and produces an enzyme that inactivates most of the antimicrobial agents (De Oliveira et al., 2000; Hoekstra et al., 2020).

The need for reliable and rapid methods for identification of *S. aureus* is crucial for the control of the disease and for economically sound udder health management. The identification and characterization of *Staphylococci* can be performed using various phenotyping and genotyping techniques such as biochemical investigations, molecular assays including polymerase chain reaction (PCR), DNA sequencing (ribosomal gene and complete genome sequencing), and physical techniques such as matrix‐assisted laser desorption/ionization time‐of‐flight mass spectrometry (Bianchi et al., 2014; Barreiro et al., 2017; Phuektes et al., 2003). Molecular methods provide accurate confirmation of the identity of microorganism isolated from mastitis (SCM or CM) milk samples (Annamanedi et al., 2021; Hoque et al., 2018). In most laboratories of Bangladesh, characterization of *Staphylococci* is done by the identification of phenotypic traits of cultured bacteria, which sometimes provide confusing results. Again, it needs sufficient time for bacterial growth in culture medium. Conversely, PCR‐based approach is a rapid, efficient and cost‐effective tool for accurate characterization of the causal agents (Bianchi et al., 2014). A series of reports are available describing the use of PCR and ribosomal (16S rRNA) gene sequencing to identify and characterize *Staphylococcal* isolates (Bianchi et al., 2014; Phuektes et al., 2001; Shome et al., 2011). Moreover, multiplex PCR (mPCR) assay for simultaneous detection of SCM causing pathogens could be a promising tool for monitoring mastitis at the herd level (Ashraf et al., 2017; Charaya et al., 2015). For rapid and reliable detection of *S. aureus* and its enterotoxins in milk, a number of mPCR assays have been established (Ashraf et al., 2017; Rahman et al., 2018; Charaya et al., 2015).

**FIGURE 1:**
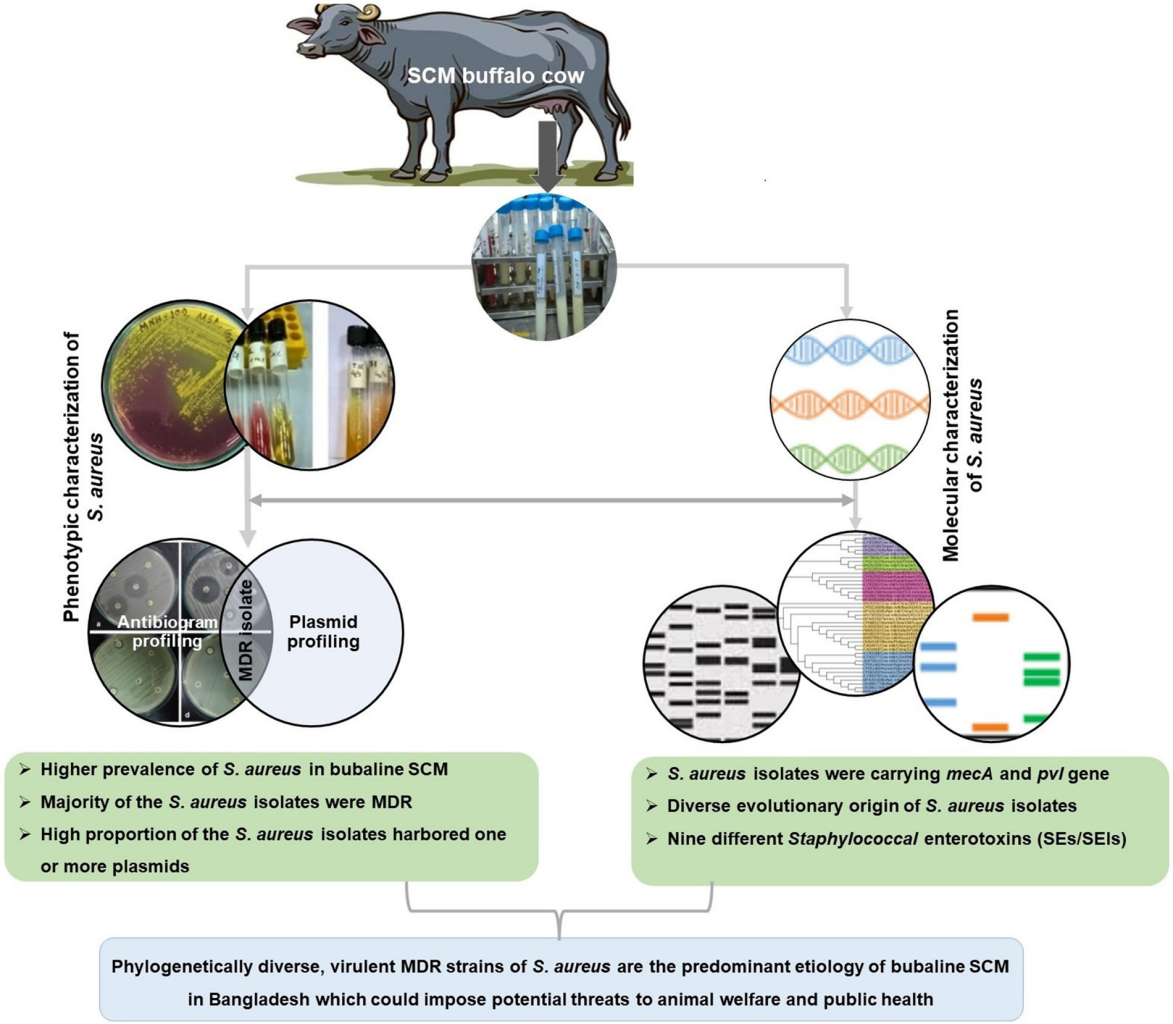
Schematic representation of subclinical mastitis (SCM) study in buffalo cows. The research works include both phenotypic (selective culture and biochemical tests) identification, antibiogram and plasmid profiling, and molecular (16S rRNA gene sequencing, marker specific multiplex PCR) characterization of *Staphylococcus aureus* virulence and enterotoxin gene(s) in milk of SCM buffaloes. Phylogenetically diverse, virulent, multidrug resistant (MDR) *S. aureus* strains are the predominant aetiology of SCM in buffalo cows.

Buffalo in Bangladesh is mainly indigenous in origin and most of them are riverine type with exception of some swamp type in eastern part of the country (Hoque et al., 2011). Most of the buffalo farmers either commercial or household small‐scale farmers of Bangladesh fail to understand the consequences of SCM pathogens on udder health and how these microorganisms affect overall milk production and milk quality. Hence, reliable information on the prevalence of *S. aureus* and other *Staphylococci* among buffalo cows in Bangladesh is limited. However, no detailed studies on the genetic information of *S. aureus*, its antibiogram, and virulence gene(s) profiling in mastitis samples of cows and buffaloes are available in Bangladesh. This study therefore utilized an mPCR protocol with 11 primer sets to simultaneously identify *S. aureus*, two virulence marker genes and nine *Staphylococcal* enterotoxin (SE) genes in *S. aureus* isolated from SCM milk of buffalo cows (Figure 1).

## 2 MATERIALS AND METHODS

### 2.1 Buffalo population

The present study was conducted on 125 lactating riverine buffalo cows randomly selected from 38 smallholders (at least two buffalo cows per farm) and two small scale commercial farms (>30 buffalo cows per farm) of Chattogram (22.3569° N, 91.7832° E), Mymensingh (24.7471° N, 90.4203° E), Lakshmipur (22.9447° N, 90.8282° E), Bhola (22.6855° N, 90.6439° E), and Satkhira (22.3155° N, 89.1115° E) districts of Bangladesh (Figure S1). The buffaloes were of 4 to 9 years of age, 2.5–3.5 body condition score (BCS), and 2–4 parity. The average milk production was 7.5 L per day. Among these, 75 buffalo cows belonged to smallholder farms, whereas 50 cows belonged to two commercial farms. Due to the absence of observable clinical signs, presumptive diagnosis of SCM was made by California Mastitis Test (CMT) following our previously published protocol (Hoque et al., 2015) and manufacturer's instruction (CMT^®^, Original Schalm reagent, ThechniVet, USA) (Figure S2). Buffalo cows showing clinical signs of mastitis were excluded from the study.

### 2.2 Collection of milk samples and bacteriological examination

Five hundred quarter milk samples were collected from 125 riverine buffalo cows. Before collection, quarters were soaked with 70.0% ethanol and dried off using tissue paper. Initially, one to two drops of milk were discarded and then 10–15 ml of milk from each of the four quarters (left front; LF, left hind; LH, right front; RF, and right hind; RH) was collected aseptically in sterile plastic tubes (15 ml Falcon Tube^®^). Immediately after collection, milk samples were subjected to CMT to diagnose quarters with SCM (Figure S2). After on farm screening, the SCM‐positive samples (*n* = 188) were kept in icebox (4–8°C) and transported to the laboratory for further bacteriological examination and molecular analysis.

The culture‐based bacteriological isolation and identification was performed according to National Mastitis Council (Nickerson et al., 2004) standards (Hoque, Istiaq, Clement, et al., 2020; Reddy et al., 2014). In brief, collected SCM milk samples were subjected to selective isolation and identification of *S. aureus*, *Streptococcus agalactiae*, *Streptococcus uberis*, *Streptococcus dysgalactiae*, *Escherichia coli*, *Klebsiella* spp., Coagulase Negative *Staphylococci* (CNS), *Enterobacter* spp., and *Bacillus* spp. according to previously published methods (Cheng et al., 2019; Hoque et al., 2018; Hoque, Istiaq, Clement, et al., 2020). These microorganisms were identified based on their colony morphology, hemolytic patterns on blood agar, and Gram‐staining (Cheng et al., 2019). Gram‐positive bacteria were further confirmed based on their biochemical characteristics in indole, methyl red, Voges‐Proskauer (VP), catalase, oxidase, urease and triple sugar iron (TSI) tests, and growth on mannitol salt agar. Gram‐negative bacteria were confirmed based on the results of indole, methyl red, citrate (IMViC) tests and lactose fermentation on MacConkey agar (Gomes et al., 2016; Hoque et al., 2018; Hoque, Istiaq, Clement, et al., 2020). Finally, all isolates were stored at −80°C for further genomic identification.

### 2.3 DNA extraction from *S. aureus* isolates

Genomic DNA was extracted from colonies of overnight cultures with typical growth of *Staphylococci* on Mannitol salt agar (MSA, Oxoid, Hampshire, UK) using the boiled method (Hoque, Istiaq, Clement, et al., 2020). In brief, pure bacterial culture from nutrient agar was subcultured in nutrient broth. Each millilitre broth culture was taken in separate Eppendorf tube and centrifuged at 10,000 rpm for 5 min. The supernatant was discarded and any remaining liquid was removed by soaking (with wipes). The pellet was collected and replenished with 200 µl autoclaved deionized water followed by finger shaking to dissolve the pellet. The Eppendorf tubes were then placed in a water bath at 100°C for 10 min. Immediately after boiling, the Eppendorf tube was kept in ice for 10 min followed by centrifugation at 10,000 rpm for 10 min. Finally, about 100–150 µl supernatant containing bacterial chromosomal DNA was collected. DNA quantity and purity were determined with NanoDrop 2000 (ThermoFisher, USA) by measuring 260/280 absorbance ratios, and stored at −20°C.

### 2.4 PCR amplification, 16S rRNA gene sequencing, and data analysis

Following DNA extraction, PCR amplification was carried out targeting the ribosomal (16S rRNA) gene fragments. The 16S rRNA gene was amplified using universal primers 27F (5´‐AGAGTTTGATCCTGGCTCAG‐3´) and U1492R (5´‐ CTACGGCTACCTTGTTACGA‐3´) (Masomian et al., 2016). Agarose gel electrophoresis (1.2% wt/vol) was used to verify the presence of PCR products. DNA purification and standard Sanger sequencing were conducted for five isolates following the instructions of the sequencing company (Macrogen Inc. Seoul, Korea). Using Molecular Evolutionary Genetics Analysis (MEGA) version 7.0 for the larger datasets (Kumar et al., 2016), the nucleotide sequence of the corresponding isolates was visualized. In order to search for nucleotide sequences similarity, Genbank databases were used by online program nucleotide BLAST (http://www.ncbi.nlm.nih.gov/blast/Blast.cgi) (Donkor et al., 2014). Closely related sequences were retrieved from NCBI and subjected to multiple sequence alignment by ClustalW program (Thompson et al., 2003). Trimmomatic program (version 0.39) was used to estimate the quality of each sequence, edit and trim poor‐quality sequences (Rahman et al., 2021). A maximum‐likelihood tree was generated by MEGA 7.0 software using default parameters, and visualized by iTOL v5.6.1 (Letunic & Bork, 2019). Nodal confidence in the resulting phylogenetic relationships was assessed using the bootstrap test (1000 replicates). The sequences were submitted in NCBI (https://www.ncbi.nlm.nih.gov), with the accession numbers from ON386175 to ON386179.

### 2.5 Antibiogram profiling

The in vitro antibiogram profile of 109 *S. aureus* isolates was determined using the disk diffusion method following the Clinical Laboratory Standards Institute (CLSI) 2017 guidelines (Arendrup et al., 2017). Antibiotics were selected for susceptibility testing corresponding to a panel of antimicrobial agents (Oxoid, Hampshire, UK) of interest to the dairy industry and public health in Bangladesh. The selected groups of antibiotics included penicillins (ampicillin, 10 µg/ml), tetracyclines (doxycycline 30 µg/ml; tetracycline, 30 µg/ml), nitrofurans (nitrofurantoin, 300 µg/ml), quinolones (ciprofloxacin, 10 µg/ml; nalidixic acid, 30 µg/ml), cephalosporins (cefoxitin, 30 mg/ml; cefotaxime, 30 µg/ml; cefazolin, 30 µg/ml), penems (imipenem, 10 µg/ml), phenols (chloramphenicol, 30 µg/ml), aminoglycosides (gentamycin, 10 µg/ml), and macrolides (erythromycin, 15 µg/ml; azithromycin, 15 µg/ml). Phenotypic AMR was obtained using a microdilution method for antimicrobials commonly administered to dairy cattle and humans, following CLSI guidelines 2017 (CLSI, 2017).

### 2.6 PCR amplification of *tsst‐1* and *pvl* toxin encoding genes in *S. aureus* isolates

The genomic DNA extracted from the *S. aureus* isolates (as mentioned above) was used to screen for the toxic shock syndrome toxin‐1 (*tsst*‐1) gene and the Panton‐Valentine leukocidin (*pvl*) toxin gene following previously described protocols (Haran et al., 2012). PCR products were electrophoresed in a 1.5% agarose gel containing 500 µg ml^–1^ of ethidium bromide, and the gel was visualized by UV transilluminator.

### 2.7 Multiplex PCR for detection of enterotoxin (SEs/SEls) genes in *S. aureus* isolates

A modified mPCR was used for the detection of enterotoxin genes from the *S. aureus* isolates (Bianchi et al., 2014; Tarekgne et al., 2016). In brief, each mPCR reaction was performed with a final reaction volume of 50 µl. It was composed of 45 µl of reaction mixture containing a final concentration of 1× AmpliTaq buffer, 4 mM MgCl2, 2 U of AmpliTaq Gold polymerase (Applied Biosystems, Foster City, CA, USA), 400 µM deoxynucleoside triphosphates (dNTPs; New England Biolabs, Beverly, MA), and 300 nM each SE primer and 60 nM 16S rRNA primers. Finally, 5 µl of DNA (10 ng/µl) was added to the mixture. The cycling condition for PCR amplifications was as follows: 95°C for 15 min, 68°C for 45 s, 72°C for 1 min, 35 cycles of 1 min at 95°C, 64°C for 45 s, 72°C for 1 min, and a final extension at 72°C for 15 min. Amplified PCR products were visualized on 1.5% agarose gel prepared in 1× TAE buffer. After gel electrophoresis, the images were captured using Image ChemiDoc™ Imaging System (Bio‐Rad, USA). The *S. aureus* isolates were investigated for the presence of genes coding for nine selected SEs (sea, seb, sec, sed, see, seg, seh, sei, ser) and two SEls (selj, selp) according to the recommended guidelines of the European Union Reference Laboratory for coagulase‐positive *Staphylococci* by using two independent mPCR reaction protocols (Bianchi et al., 2014). Primers for *sed*, *see*, *seg*, *sei*, and *tsst‐*1 were combined in reaction mixture 1 and primers for *sea*, *seb*, *sec*, *seh*, *sej*, and 16S rRNA were combined in reaction mixture 2 (Carfora et al., 2015). The primers used were supplied by Invitrogen Life Technologies (Carlsbad, CA, USA) and listed in Table S1. Each *S. aureus* isolate positive for at least one of the “classical SEs” coding gene by mPCR was tested for *Staphylococcal* enterotoxins production (SEA‐SED), performed by the RPLA method (Carfora et al., 2015), using the kit SET‐RPLA (TD 9000; Oxoid, Hampshire, UK) according to the manufacturer's instructions.

### 2.8 Plasmid extraction

All of the *S. aureus* isolates were further tested for the presence of plasmid. Plasmid DNA from *S. aureus* isolate was extracted according to previously published methods (Hoque et al., 2018; Tumlam et al., 2013). Briefly, a single colony of pure *S. aureus* was inoculated into 5 ml of Luria‐Bertani (LB) broth (Oxoid, Hampshire, UK) and incubated in a shaking incubator at 200 rpm at 37°C for 16–18 h, and then centrifuged at 4800 rpm for 5 min, and the resulting cell pellets were resuspended in 300 µl, TENS buffer (Tris‐EDTA‐NaOH/SDS). The solution was mixed for 2–3 s until the mixture became sticky. The samples were incubated in ice for 10 min to prevent the degradation of chromosomal DNA. Thereafter, 150 µl 3 M sodium acetate (Sigma‐Aldrich, USA, pH 5.2) was added and vortexed 2–5 s to mix completely. The mixture was rotated again at 13,200 rpm for 10 min to pellet cell debris and chromosomal DNA. The supernatant was transferred into a fresh microtube and mixed with 1 ml of 95% EtOH (Ethanol) precooled at −20°C and further spun for 2 min to pellet plasmid DNA and RNA. The supernatant was also discarded, and the pellet rinsed twice with 500 µl of 70% EtOH and dried at room temperature. For the subsequent steps, the isolated plasmid DNA was resuspended in Tris‐EDTA buffer; at pH 8 and 200 ng/µl RNAse were also added. Plasmids were separated by electrophoresis in 1.5% agarose gel containing 500 µg ml^–1^ of ethidium bromide and the gel was visualized by UV transilluminator at a voltage of 4.5 V/cm; buffer: 1x TAE (TrisAcetate‐EDTA); time: 3 h, and thereafter observed under UV light to visualize the bands properly. The image was recorded and analyzed using Image Master VDS (Pharmacia Biotech).

### 2.9 Statistical analysis

Data were entered into Microsoft Excel 2016^®^ (Microsoft Corp., Redmond, WA, USA) and analyzed using Excel and SPSS version 20 (IBM Corp., Armonk, NY, USA). The Cochran–Mantel–Haenszel *χ* ^2^ test was performed to compare *S. aureus* mastitis between the studied dairy farms and areas, and also to compare the cultural results in PCR‐positive and PCR‐negative samples. A prevalence percentage was calculated by dividing the number of positive samples for the given category by the total samples tested within that category. The prevalence formula was applied for determining prevalence percentage of SCM and *S. aureus*. The AMR patterns, resistance, intermediate, and sensitivity were calculated using the CLSI (2017) guideline using the cut‐off as provided in the brochure of the manufacturer (Oxoid, Hampshire, UK). For the test, *p* < 0.05 was considered statistically significant.

## 3 RESULTS

### 3.1 Prevalence and aetiology of subclinical mastitis

**FIGURE 2:**
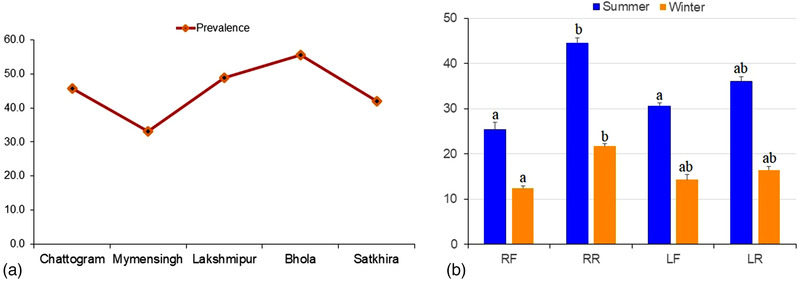
Subclinical mastitis (SCM) in riverine buffalo cows. (a) Prevalence of SCM caused by *Staphylococcus aureus* in the study areas. (b) Quarter‐wise prevalence of *S. aureus* in the SCM milk of riverine buffalo cows during summer and winter seasons.

**FIGURE 3:**
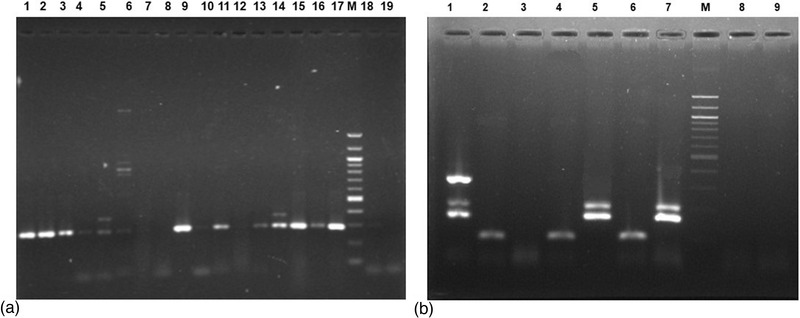
The agarose gel electrophoresis image of *Staphylococcus aureus*‐specific polymerase chain reaction in isolates of bubaline subclinical mastitis (SCM) milk. (a) Visualization of gel image through ultraviolet radiation. Lane 1: positive control *S. aureus* M33C2; lanes 2–15: representative *S. aureus* isolates with positive isolates at 2nd, 3rd, 9th, 11th, 13–15th lanes; lane 16: positive control *S. aureus* M13; lane 17: positive control *S. aureus* ATCC 25923; lane M: molecular size marker (100 bp DNA ladder); lane 18: *S. epidermidis* and lane 19: reagent blank. (b) Visual of gel through ultraviolet radiation showing *mecA* gene. Lane 1: positive control *S. aureus* M33C2; lanes 2–6: representative *S. aureus* isolates with *mecA* positive isolates at 2nd, 4–6th; lane 7: positive control *S. aureus* ATCC 25923; lane M: molecular size marker (100 bp DNA ladder); lane 8: negative control *S. epidermidis* and lane 9: reagent blank.

A total of 500 quarter milk samples from 125 riverine buffalo cows were screened through CMT to diagnose buffalo cows with SCM. The CMT found 188 quarter milk samples (37.6%) positive for SCM. Among these SCM‐positive milk samples, through selective culture and biochemical analysis, 291 isolates of bacteria were obtained, and of them, 37.4% (109/291) isolates were found to be harbouring *S. aureus*. From the culture‐positive samples, a total of 633 bacteria of nine genera were isolated. Among these bacteria, *S. aureus* was the single most aetiology of SCM (37.4%) in buffalo cows followed by *E. coli* (7.6%), *S. agalactiae* (6.2%), *Klebsiella* spp. (4.5%), coagulase‐negative *Staphylococci* (CNS) (4.1%), *S. uberis* (3.8%), *S. dysagalactiae* (3.1%), *Bacillus* spp. (2.4%), and *Enterobacter* spp. (1.4%). Mixed infections were found in 29.5% culture‐positive quarters and of them, *Staphylococci* and *Streptococci* were the most common mixed causes of bubaline SCM (28.8%) in Bangladesh (Table 1). In our present study, there were marked differences in the prevalence of *S. aureus* in the SCM milk across the study areas, and the highest prevalence was recorded in Bhola (55.6%) followed by Lakshmipur (48.8%), Chittagong (45.7%), Satkhira (42.0%), and Mymensingh (33.1%) (Figure 2a). Comparing the seasonal effect on the SCM caused by *S. aureus*, our analysis revealed significant variations (*p* < 0.05) in the prevalence of *S. aureus* in the milk of SCM cows. The quarters of the buffalo cows were more prone to *S. aureus* in summer season (March to September) than the winter months (October to February). We also found significant variations (*p* < 0.05) in the prevalence of *S. aureus* according to the quarter position of the buffalo cows. In this study, the highest prevalence of *S. aureus* isolates was found in RR (44.5%) quarters, followed by LR (36.0%), LF (30.5%), and RF (25.4%) quarters (Figure 2b). Amplification with genus specific PCR assay successfully confirmed the isolates as *S. aureus* by amplification of DNA fragments ranging in size from 100 to 300 bp (Figure 3a). The PCR amplification of *mecA* gene was done for all isolates to detect the species of *S. aureus*. In this study, the PCR product appeared as a single band DNA with a size equal to 163 bp fragment corresponding to the *mecA* amplicon (Figure 3b).

**TABLE 1:** The relative distribution of bacterial aetiologies identified from quarter milk samples of buffalo cows infected with subclinical mastitis

Isolated bacteria	No. of quarters	No. of isolates	Percentage (%)
Major pathogens			
*Staphylococcus aureus*	76	109	37.4
*Streptococcus agalactiae*	11	18	6.2
*Streptococcus uberis*	7	11	3.8
*Streptococcus dysgalactiae*	6	9	3.1
*Escherichia coli*	13	22	7.6
*Klebsiella* spp.	7	13	4.5
Minor pathogens			
Coagulase Negative Staphylococci (CNS)	9	12	4.1
*Bacillus* spp.	4	7	2.4
*Enterobacter* spp.	3	4	1.4
Mixed infections*	52	86	29.5
Total	188	291	100.0

* *Staphylococci* + *Streptococci* (15), *Staphylococci* + *E. coli* (11), *Staphylococci* + *Bacilli* (10), *Streptococci* + *E. coli* (9), *Streptococci* + CNS (7).

### 3.2 Phylogenetic analysis reveals diverse evolutionary origin of *S. aureus* strains

**FIGURE 4:**
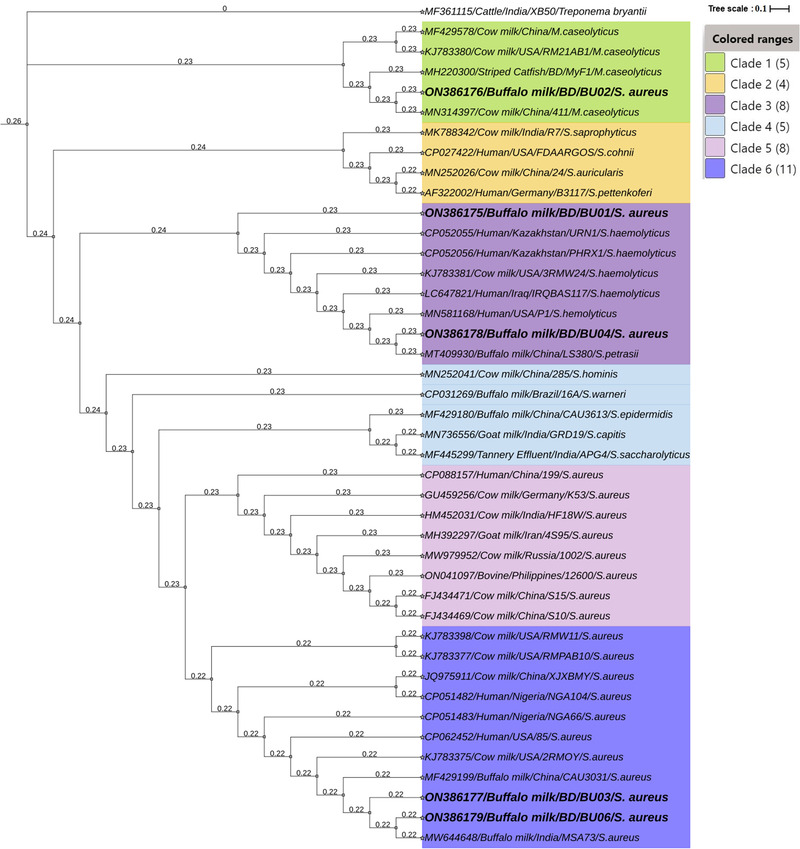
Phylogenetic tree of 16S rRNA gene sequences showing the position of *Staphylococcus aureus* strains ON386175–ON386179 (Buffalo milk/BD/BU01–BU05) isolated from subclinical mastitis milk of riverine buffalo cows in Bangladesh. *Treponema bryantii* MF361115 is used as an outgroup. The sequences were aligned with the ClustalW program and were constructed with the maximum‐likelihood algorithm and the Kimura 2‐parameter model integrated in the MEGA 7.0 program. Finally, the tree was visualized using the interactive tree of life (iTOL) webserver (https://itol.embl.de/personal_page.cgi). The GenBank accession numbers, source, host, and countries of origin of the sequences (*n* = 41) are placed before the name of the corresponding species. Bootstrap values are presented next to the tree. The scale bar indicates the number of nucleotides substitutions per site.

The isolated 16S rRNA gene sequences of the bubaline SCM‐associated *S. aureus* have shown 99% compatibility with other reference sequences retrieved from the NCBI used to build the phylogenetic tree. Analysis of the 16S RNA genes of the *S. aureus* isolates (*n* = 5) from bubaline SCM cases revealed that Bangladeshi *S. aureus* strains diverged into three distinct clades (Clade 1, Clade 3, and Clade 6) and showed close evolutionary relationship with some strains that are available in GenBank database of NCBI (Figure 4). Moreover, the phylogenetic tree diverged into six clades, and all of the Bangladeshi *S. aureus* strains clustered with strains from different countries. In the phylogenetic tree, Clade 1 (five lineages) branched as first or as the most ancient divergence of *S. aureus* where the 16S rRNA sequence of ON386176/Buffalo milk/BD/BU02/*S. aureus* strain closely clustered with different strains of *Macrococcus caseolyticus* originated from cow milk belonged to China (MN314397) and the United States (KJ783380). The second clade (Clade 2) consisted of four lineages, and none of them showed homology with our sequences. The next clade of *S. aureus* (Clade 3; eight lineages) to diverse is consisted of two Bangladeshi isolates (ON386175/Buffalo milk/BD/BU01 and ON386178/Buffalo milk/BD/BU04) which showed close relationship with *Staphylococcus petrasii* MT409930 strain of buffalo milk of China and *S. haemolyticus* KJ783381 strain of cow milk sequenced from the United States (Figure 4). The sixth phylogenetic clade (Clade 6; 11 lineages) is consisted of two Bangladeshi *S. aureus* strains (ON386177/Buffalo milk/BD/BU03 and ON386179/Buffalo milk/BD/BU06), and branched together, representing divergence from a common ancestor. These two *S. aureus* strains showed close phylogenetic relatedness with two *S. aureus* strains originated from buffalo milk of India (MW644648) and China (MF429199), and that of cow milk from China (JQ975911) and the United States (KJ783375, KJ783397, KJ783398) (Figure 4). Notably, among these five strains of *S. aureus* causing SCM in buffalo cows, four strains (ON3861175, ON3861177, ON3861178, ON3861179) had a close phylogenetic relationship with different pathogenic *Staphylococcal* species (including *S. aureus*) of humans reported from United States (CP062452, MN581168), Nigeria (CP051482, CP051483), Iraq (LC647821), and Kazakhstan (CP052055, CP052056) indicating their potential zoonotic significance (Figure 4).

### 3.3 Subclinical mastitis associated *S. aureus* showed multidrug resistance properties

*Staphylococcus aureus* revealed varying susceptibility to 15 antibiotics belonging to nine different groups (Table 2). Out of 109 *S. aureus* isolates tested, 105 (96.33%) isolates were resistant to antibiotics belonging to more than one group of antibiotics tested and were classified as MDR isolates (Table 2). The antibiogram profiling revealed that *S. aureus* showed the highest resistance rate towards ampicillin (92.7%, 101/109) with a breakpoint measuring less than 28 mm followed by doxycycline (90.8%, 99/109), tetracycline (80.7%, 83/109), chloramphenicol (70.6%, 77/109), ciprofloxacin (59.6%, 65/109), nitrofurantoin (56.0%, 65/109), and to a less extent to, cefotaxime (48.6%), and gentamicin (42.2%) with different breakpoints (≤23 mm). Likewise, the *S. aureus* isolates demonstrated intermediate resistance against imipenem (63.3%), cefotaxime (44.9%), cefoxitin (40.4%), and gentamicin (37.6%) (Table 2). Conversely, the *S. aureus* isolates demonstrated the highest susceptibility to azithromycin (80.7%) followed by erythromycin (60.5%), cefazolin (56.9%), nalidixic acid (53.2%), and cefoxitin (48.6%). However, none of the *S. aureus* isolate was resistant to azithromycin (Table 2).

**TABLE 2:** Antibiogram profile of *S. aureus* isolates (N = 109) against commonly available antibiotics

Antibiotic	Disc potency (µg)	Breakpoint to declare resistance (≤mm)	Sensitive	Intermediate	Resistance
			% (No.)	% (No.)	% (No.)
AMP	10	28	0 (0)	7.3 (8)	92.7 (101)
DOX	30	23	0 (0)	9.2 (10)	90.8 (99)
CTX	30	13	6.4 (7)	44.9 (49)	48.6 (53)
TE	30	23	9.2 (10)	14.7 (16)	80.7 (83)
CHL	30	12	10.1 (11)	19.3 (21)	70.6 (77)
CIP	10	20	12.8 (14)	27.5 (30)	59.6 (65)
IMP	10	22	28.4 (17)	63.3 (69)	21.1 (23)
NIT	10	64	19.3 (21)	24.8 (27)	56.0 (61)
GEN	10	12	20.2 (22)	37.6 (41)	42.2 (46)
CFX	30	24	48.6 (53)	40.4 (44)	11.0 (12)
NAL	30	16	53.2 (58)	31.2 (34)	15.6 (17)
CZ	30	20	56.9 (62)	26.6 (29)	16.5 (18)
ERY	15	20	60.5 (66)	30.3 (33)	9.2 (10)
AZ	15	20	80.7 (88)	19.3 (21)	0 (0)

Abbreviations: AMP, ampicillin; AZ, azithromycin; CFX, cefoxitin; CHL, chloramphenicol; CIP, ciprofloxacin; CTX, cefotaxime; CZ, cefazolin; DOX, doxycycline; ERY, erythromycin; IMP, imipenem; GEN, gentamicin; NAL, nalidixic acid; NIT, nitrofurantoin; No., number of isolates; TE, tetracycline.

**FIGURE 5:**
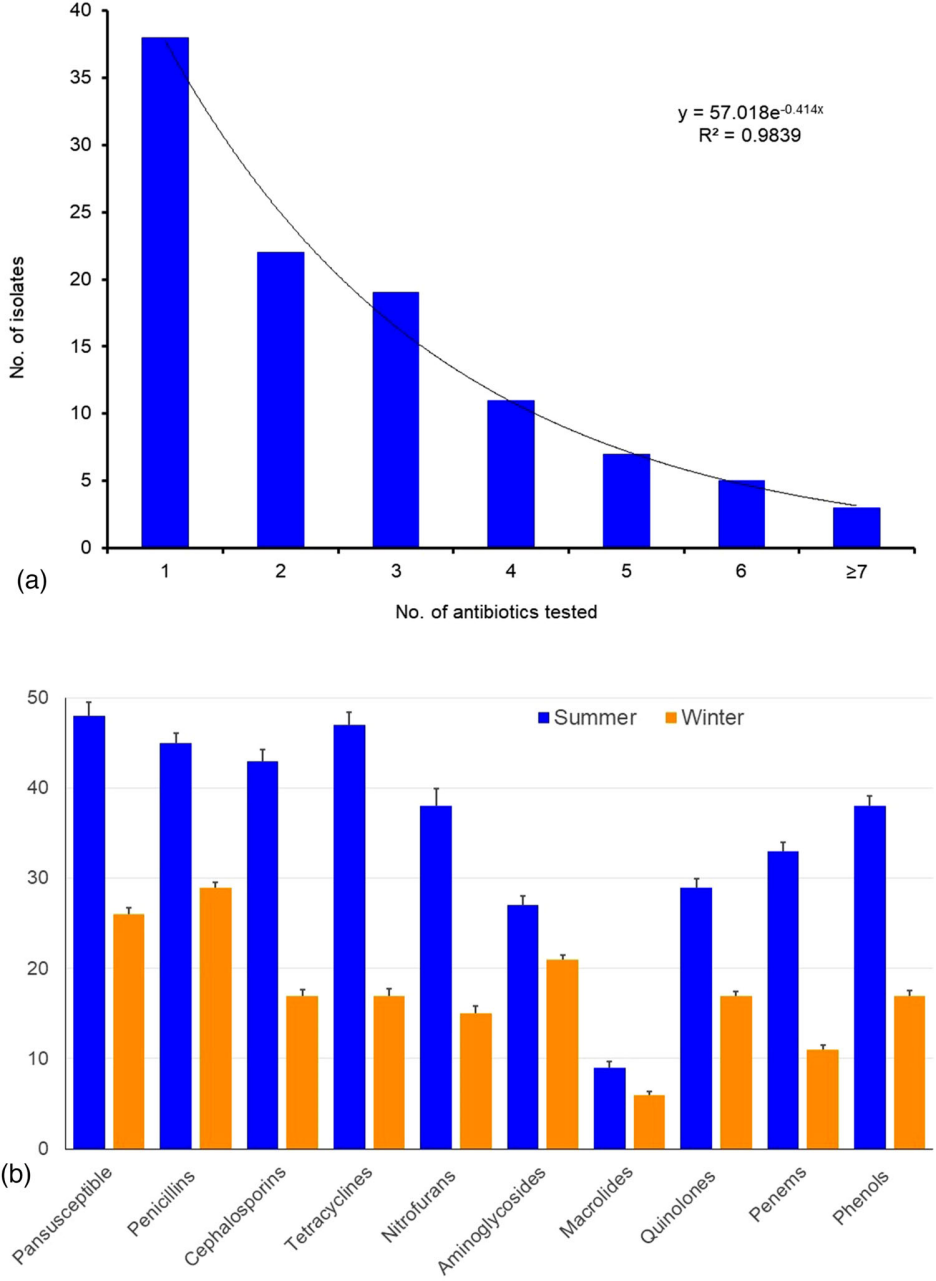
Antibiogram profiling: (a) frequency distribution of antimicrobial agent resistance patterns of *Staphylococcus aureus* isolates. (b) Distribution of antimicrobial resistance to different classes of antimicrobials in *S. aureus* isolates during summer and winter season. Antimicrobial resistance for all *S. aureus* isolates was determined by the Kirby Bauer method with individual antibiotic discs.

The resistance profile of *S. aureus* isolates differed significantly (*p* < 0.05) by the tested groups of antibiotics. Comparing the MDR phenomena of *S. aureus*, we found that 38 (34.9%) isolates were resistant to one class of antibiotic, 22 (20.2%) were resistant to two classes of antibiotics, 45 (41.3%) were resistant to three or more classes of antibiotics, and thus MDR (Figure 5a). Of the 109 isolates detected, 74 (67.9%) were pansusceptible, and the proportion isolates and their MDR properties varied significantly (*p* < 0.05) between the two seasons and always remained higher during summer season (Figure 5b). The *S. aureus* isolates showed higher MDR phenomena against tetracyclines (63.5%) followed by penicilins (60.8%), cephalosporins (58.1%), nitrofurans (51.4%), and phenols (51.4%). However, the MDR properties of *S. aureus* isolates were against macrolides (12.1%) (Figure 5).

### 3.4 Prevalence of *mecA*, *pvl*, and enterotoxin (SE/SEl) genes

The toxin gene profile of *S. aureus* isolates recovered from SCM milk samples of buffalo cows is listed in Table 3. All the 109 *S. aureus* isolates were tested by mPCR for the presence of *mecA*, *pvl* and SEs/SEls genes. Nine different toxinotypes were identified in the tested isolates along with *mecA* and *pvl* genes. In this study, 26.6% and 14.7% *S. aureus* isolates harboured the *mecA* and *pvl* gene, respectively. The SEs/SEls genes most frequently detected were *sea* which was prevalent in 11.9% of the isolates, followed by *sec* (7.4%), *sed* (4.6%), *seg* (3.7%), and *seh* (3.7%). None of the isolates harboured *see* and *selp* genes. Toxinotypes composed of a single gene were present in 72.48% (79/109) isolates, while only 27.52% (30/109) isolates harboured more than one toxin gene, exhibiting a significant heterogeneity (Table 3). Overall, 100 isolates out of 109 (91.7%) were found positive for the presence of SEs coding genes (*sea*, *seb*, *sec*, or *sed*) by mPCR (Table 4), and all were tested for enterotoxin production by the SET‐RPLA kit. Overall, 82 colonies out of 100 positive colonies were found to be positive for SEs and some colonies presented more than one SE gene simultaneously, for a total of 148 PCR positive results. The in vitro expression of classical enterotoxins revealed that *sed* was the mostly expressed (93.1%) SEs followed by *sea* (82.8%), *sec* (75.9%), and *seb* (31.0%) (Table 4). However, the *eta*, *etb* and *tsst*‐1 genes were not detected in any isolate.

**TABLE 3:** *Staphylococcal* enterotoxin (SEs/SEls) gene profile (toxinotypes) of the 109 *S. aureus* isolates in milk from bubaline subclinical mastitis

Toxin gene profiles	No. (%) of positive quarters	Total no. (%) of positive isolates
*mecA*	44 (23.4)	29 (26.6)
*pvl*	28 (14.9)	16 (14.7)
*sea*	21 (11.2)	13 (11.9)
*sec*	15 (8.0)	8 (7.4)
*sed*	9 (4.8)	5 (4.6)
*seg*	7 (3.7)	4 (3.7)
*seh*	5 (2.7)	4 (3.7)
*sea*, *sei*	8 (4.2)	3 (2.7)
*seb*, *sec*	6 (3.2)	3 (2.7)
*sea*, *sed*, *selj*	12 (6.3)	7 (6.4)
*sea*, *sed*, *ser*	10 (5.3)	5 (4.6)
*sec*, *seg*, *sei*	8 (4.2)	4 (3.7)
*sed*, *seg*, *sei*	7 (3.7)	3 (2.7)
*sea*, *sed*, *seg*, *selj*	8 (4.2)	5 (4.6)
Total	188 (100.0)	109 (100.0)

**TABLE 4:** In vitro expression of classical enterotoxins (*sea*, *seb*, *sec*, *sed*), as detected by the SET‐RPLA, in relation to the presence of corresponding genes as detected by multiplex‐PCR

Enterotoxins	No. of colonies positive for the corresponding genes	No. (%) of enterotoxin producing colonies
*sea*	29	24 (82.8)
*seb*	14	9 (31.0)
*sec*	27	22 (75.9)
*sed*	30	27 (93.1)

### 3.5 Plasmid bearing *S. aureus* isolates showed multidrug resistance

Plasmid extraction from *S. aureus* isolates revealed that 70.6% (77 out of 109) isolates were harbouring one or more plasmids. From the result of antimicrobial susceptibility test, it has been found that 42.2% (46/109) of these plasmid bearing isolates were MDR isolates. The remaining 31 plasmids were susceptible to all the tested antibiotics. The size of the plasmids was found to be >10 kb. No plasmid DNA was found in 29.4% (32/109) isolates, and among these isolates, 19 (59.4%) were resistant to at least three or more antimicrobials.

## 4 DISCUSSION

Subclinical mastitis is considered one of the most infectious diseases that affect dairy animals with an annual prevalence of 37% (El‐Ashker et al., 2015). Although epidemiological prevalence of *S. aureus* and its antimicrobial resistance pattern have been extensively studied in livestock in other countries (Cheng et al., 2019; El‐Ashker et al., 2015; Zayda et al., 2020), limited studies with particular emphasis to SCM in lactating dairy cows and buffaloes have been carried out in Bangladesh. Therefore, this study aimed to characterize *S. aureus* isolates in milk obtained from riverine buffalo cows affected with SCM to their antimicrobial resistance and virulence gene(s) under the current farming situation of Bangladesh. Moreover, the evolutionary relationship between the strains isolated from bubaline SCM milk and those retrieved from the global database (NCBI) belonging to cow and buffalo milk was also investigated (Figure 1).

The prevalence of SCM in riverine buffaloes was 37.6% which is consistent with many of the studies from our neighbouring countries India (25.0%–35.0%) (Krishnamoorthy, Goudar, et al., 2021; Srinivasan et al., 2013) and Pakistan (35.0%–45.0%) (Hussain et al., 2018; Krishnamoorthy, Goudar, et al., 2021). Bacteriological examination of SCM milk samples detected *S. aureus* in 37.4% of isolates where 26.6% were MRSA isolates harbouring the *mecA* gene. This finding is in line with other investigations, where a similar prevalence of MRSA (approximately 25.0%) was observed among bovine and bubaline isolates (El‐Ashker et al., 2015; Hoque et al., 2018; Krishnamoorthy, Goudar, et al., 2021). Other dominant bacterial species detected in this study included *E. coli* (7.6%), *Streptococcus* spp. (∼10.0%), *Klebsiella* spp. (4.5%), CNS (4.1%), *Bacillus* spp. (2.4%), and *Enterobacter* spp. (1.4%). These findings on the aetiology of SCM are in agreement with the other studies conducted in various neighbouring countries (Ali et al., 2021; Pankaj et al., 2013), and elsewhere in the world (Krishnamoorthy, Suresh, et al., 2021; Osman et al., 2014). However, mixed infections were found in 29.5% culture‐positive milk samples and of them, *Staphylococci* and *Streptococci* were the most common mixed causes of bubaline SCM (∼29.0%). Similar to our findings, other researchers have also reported *S. aureus* as the main etiological agents of mastitis in different parts of India (Neelesh et al., 2010; Pankaj et al., 2013). Contrary to our findings, several earlier studies (Pizauro et al., 2019; Sampimon et al., 2009) reported a lower prevalence of *S. aureus* and a relatively higher prevalence of the environmental pathogens, such as coagulase‐negative *Staphylococci* (Zayda et al., 2020), which are formally described as a minor pathogen of mastitis .

In our present study, there were marked differences in the prevalence of *S. aureus* in SCM milk samples collected from different study areas, seasons, and quarters of the buffalo cows. The highest prevalence of *S. aureus* in SCM milk was found in Bhola (55.6%) and the lowest in Mymensingh (33.1%). Consistent with our findings, researchers from other countries have also reported that the prevalence of *S. aureus* in mastitis milk could vary in different study locations (Cheng et al., 2019; Neelesh et al., 2010; Pankaj et al., 2013; Pizauro et al., 2019). The quarter‐wise prevalence of *S. aureus* in SCM milk remained highest in right rear (44.5%) quarters and lowest in right front (25.4%) quarters. Similar prevalence of SCM in buffalo cows has been reported in India (Pankaj et al., 2013; Sharma & Maiti, 2010). The difference in prevalence of SCM and its associated pathogens found in the present and the previous studies might be because the bacteria causing mastitis are changing with topographical and management conditions. The phylogenetic tree also exhibited different lineages of *S. aureus* strains detected from SCM milk samples of buffalo cows in Bangladesh which indicated a differential evolution. The *S. aureus* isolates of the present study distributed in the three clades with those from China (MN314397, MF429578, MT409930, MF429199, JQ975911), the United States (KJ783375, KJ783380, KJ783381, KJ783397, KJ783398), and India (MW644648). Moreover, our findings provide further evidence regarding a possible transmission of human *S. aureus* strains to buffalo cows since our four isolated *S. aureus* strains had close phylogenetic relationship with human pathogenic *S. aureus* strains of different countries of the world (Figure 4). This result corroborates with our previous study in Bangladesh (Hoque, Istiaq, Clement, et al., 2020) where we reported a genetic similarity between Bangladeshi *S. aureus* isolates and *S. aureus* strains of the other countries of the world. Furthermore, several studies indicated that genetic dimension among the isolates of the world is extremely relative, and 16S rRNA analysis is considered a good discrimination approach for distinguishing unrelated isolates (Gumaa et al., 2021).

*Staphylococcus aureus* isolates were tested in vitro for their sensitivity to 15 different antibiotics that are commonly used in veterinary practices. In the current study, ampicillin (92.7%), doxycycline (90.8%), tetracycline (80.7%), chloramphenicol (70.6%), ciprofloxacin (59.6%), and nitrofurantoin (56.0%) showed the highest resistance to *S. aureus*. This was in partial agreement with the study of Ali et al. (2021). They also reported that oxytetracycline, ampicillin, and doxycycline were the most resistant antimicrobials against major mastitis pathogens like *S. aureus* (Ali et al., 2021). In addition, imipenem (63.3%), cefotaxime (44.9%), cefoxitin (40.4%), and gentamicin (37.6%) showed intermediate resistance against *S. aureus* isolates. However, the highest susceptibility to *S. aureus* isolates was found against azithromycin (80.7%), erythromycin (60.5%), cefazolin (56.9%), nalidixic acid (53.2%), and cefoxitin (48.6%). Similarly, our earlier and several other studies also reported the highest susceptibility of mastitis causing bacterial pathogens towards macrolides and aminoglycosides including azithromycin, erythromycin, cefazolin, nalidixic acid, cefoxitin, and vancomycin (Bantawa et al., 2019; Hoque, Istiaq, Clement, et al., 2020; Hoque et al., 2018; Shrestha et al., 2021). However, none of the *S. aureus* isolate was resistant to azithromycin. Indiscriminate and frequent use of these antibiotics in animals could be the reason for their ineffectiveness against mastitis causing bacterial isolates. Antibiotic resistance patterns vary among different farms, regions, states, and countries depending upon the type of organisms and use of antibiotics in particular area; therefore, antibiotic sensitivity test is suggested before the initiation of treatment. The emergence of multidrug‐resistance in MRSA is an important threat to mastitis that is resulting in the failure in treatment and control. The *S. aureus* isolates of the present study showed higher MDR properties against tetracyclines (63.5%), penicilins (60.8%), cephalosporins (58.1%), nitrofurans (51.4%), and phenols (51.4%) (Figure 5). This finding corroborates with several earlier studies which reported that MRSA always exhibits resistance to multiple antimicrobial agents, including penicillin, methicillin, oxacillin, cefoxitin, amoxicillin‐clavulanic acid, amoxicillin‐sulbactam, quinolones, macrolides, cephalosporins, tetracycline, and chloramphenicol (Algammal et al., 2020; Hoque, Istiaq, Clement, et al., 2020; Shrestha et al., 2021). The high level of AMR, including MDR, indicates that irrational use of antibiotics is practiced in the dairy farms of Bangladesh. This difference in AMR and MDR could be correlated to time and place of study as the frequency of resistance varies from time to time and place to place.

In our current investigation, a total of nine different toxinotypes were identified among the 109 isolates along with 26.6% methicillin resistant (*mecA*) and 14.7% panton‐valentine leukocidin (*pvl*) genes, and this finding is consistent with our previous study of *S. aureus* causing SCM in dairy cows (Hoque et al., 2018). A series of earlier studies through selective culture or PCR for *mecA* have reported a higher prevalence of *S. aureus* in dairy animals mastitis ranging from 16.7% to 49.6% in India, Germany, Brazil, Japan, Sweden, and China (Alba et al., 2015; Annamanedi et al., 2021; Kutar et al., 2015). The exotoxin *pvl* is one of the most important virulence factors produced by *S. aureus*, contributing to the pathogenicity of mastitis. This toxin is associated with different diseases in humans, such as pneumonia and necrotizing dermatitis (Del Giudice et al., 2009). The *pvl* gene was also identified in *S. aureus* strains isolated from cases of mastitis (Hoque et al., 2018; Martins et al., 2015). However, this is the first report showing the expression of this toxin in *S. aureus* isolated from SCM of buffalo cows in Bangladesh. The *Staphylococcal* enterotoxin (SEs/SEls) genes most frequently detected were *sea* (11.9%), *sec* (7.4%), *sed* (4.6%), *seg* (3.7%), and *seh* (3.7%). Toxinotypes composed of a single gene were present in 72.48% and more than one toxin genes in 27.52%, exhibiting a significant heterogeneity. In this study, 91.7% *S. aureus* isolates were found positive for the classical expression of SEs coding genes (*sea*, *seb*, *sec*, or *sed*). *Staphylococcus aureus* can produce one or more exoproteins, including toxic shock syndrome toxin‐1 (*tsst‐*1), *Staphylococcal* enterotoxins (sea, seb, sec, sed, see, seg, seh, sei, and sej), the exfoliative toxins (*eta and etb*), and *pvl* (Elsayed et al., 2015; Hoque et al., 2018). On the other hand, the impact of *Staphylococcal* enterotoxins on mammary epithelial cells is still unknown. Recently, 19 serologically distinct SEs have been identified (Elsayed et al., 2015; Carfora et al., 2015; Hoque et al., 2018). Moreover, sea and seg (Carfora et al., 2015; Martins et al., 2015; Wang et al., 2009), and sec, sed, seg, and sei (Hoque et al., 2018; Martins et al., 2015; Zecconi et al., 2006) are the genes most frequently detected in *S. aureus* isolated from mastitis suffering dairy animals.

Plasmids are key reservoirs for genetic content in *Staphylococci* and allow the rapid propagation of antibiotic resistance (Mores et al., 2021). Plasmid profile of the study isolates were examined to correlate the presence of plasmid with the resistance to single or multiple drugs. Plasmid profiling revealed that 70.6% of the *S. aureus* isolates were plasmid bearing, of which 42.2% were MDR isolates showing resistance to ≥3 antimicrobials. Earlier study on AMR in *S. aureus* isolated from mastitis milk revealed that resistance genes often located on plasmids (Hoque et al., 2018; Islam et al., 2014). Thus, it could be postulated that resistance genes may exist on plasmid in *S. aureus* strains from milk samples. Since plasmids are reservoirs of and vectors for AMR in *Staphylococci* (Mores et al., 2021), plasmids could be a key factor in the pathophysiology of bubaline mastitis caused by *Staphylococci*, including MRSA.

Because of the limitations we faced with fewer samples, it would be interesting to conduct similar trials using a larger sample size with a different animal population (breed, parity, body condition, lactation) and matrices prior to undertaking a further genomic diagnostic venture to elucidate the molecular mechanisms and mutational spectra in the genome or pangenomes of *S. aureus* to better understand the pathophysiology mastitis. Another important limitation we faced in the present study was the farmers unawareness regarding the prevalence of SCM in their buffalo cows (since there were no presenting sign/clinical symptoms), and in most of the cases, SCM in buffalo cows remained undiagnosed or unattended. These limitations on mastitis in riverine buffaloes in Bangladesh are a serious problem because the inability to correctly and accurately identify the pathogen leads to difficulty in selecting the appropriate pathogen‐specific treatment or control measure to apply, consequently increasing development, and the rapid emergence of multidrug resistant *S. aureus* strains.

## 5 CONCLUSIONS

*Staphylococcus aureus* remains as the primary pathogen of concern in cases of SCM in riverine buffaloes in Bangladesh. The higher prevalence of *S. aureus* (>37.0%) in relation to other microbes (both major and minor pathogens) causing SCM in buffaloes in Bangladesh indicates a serious public health problem. Phylogenetic analysis reveals evolutionarily diverse origin of *S. aureus* isolated from SCM milk of buffaloes, and shows close relationship with many pathogenic strains of *S. aureus* of both animals and humans reported from other countries of the world. The antibiotic sensitivity tests revealed *S. aureus* as an MDR pathogen carrying both *mecA* and *pvl* genes along with nine different *Staphylococcal* enterotoxins (SEs/SEls). With limited molecular data currently available, our study provides crucial background information for future researchers and could help elucidate the epidemiological spread of *S. aureus*, the underlying molecular mechanisms, and mutational spectra in the genome or pangenomes of *S. aureus* to better understand the pathophysiology mastitis and devise optimal therapeutic strategies to curb the spread of AMR and virulence gene(s).

## AUTHORS CONTRIBUTION

*Conceptualization, supervision, writing‐original draft, data collection, formal analysis, methodology, and software*: M. Nazmul Hoque*. Conceptualization, methodology, and editing*: Anup Kumar Talukder. *Formal analysis, methodology, and software*: Otun Saha. *Formal analysis, writing‐original draft and software*: Mehedi Mahmudul Hasan. *Conceptualization, methodology, review, and editing*: Munawar Sultana. *Conceptualization, methodology, resources, and editing*: ANM Aminoor Rahman. *Conceptualization, supervision, formal analysis, methodology, and editing*: Ziban Chandra Das.

## CONFLICT OF INTEREST

The authors declare no conflict of interest.

### PEER REVIEW

The peer review history for this article is available at https://publons.com/publon/10.1002/vms3.942

## ETHICS STATEMENT

The authors confirm that the ethical policies of the journal, as noted on the journal's author guidelines page, have been adhered to and the appropriate ethical review committee approval has been received. All efforts were made considering the welfare of the experimental animals.

## Supporting information

Supplementary Information

Supplementary Information

Supplementary Information

Supplementary Information

## Data Availability

The 16S rRNA gene sequence data reported in this article have been deposited in the NCBI database and are available under GenBank accession numbers: ON386175 to ON386179 (https://submit.ncbi.nlm.nih.gov/subs/?search=SUB11406341).
