# Supplementary figures and images for: Antibiogram and virulence profiling reveals multidrug resistant Staphylococcus aureus as the predominant aetiology of subclinical mastitis in riverine buffaloes
Source: Vet Med Sci. 2022 Sep 22;8(6):2631–45. doi: 10.1002/vms3.942 (PMC9677375; doi:10.1002/vms3.942)

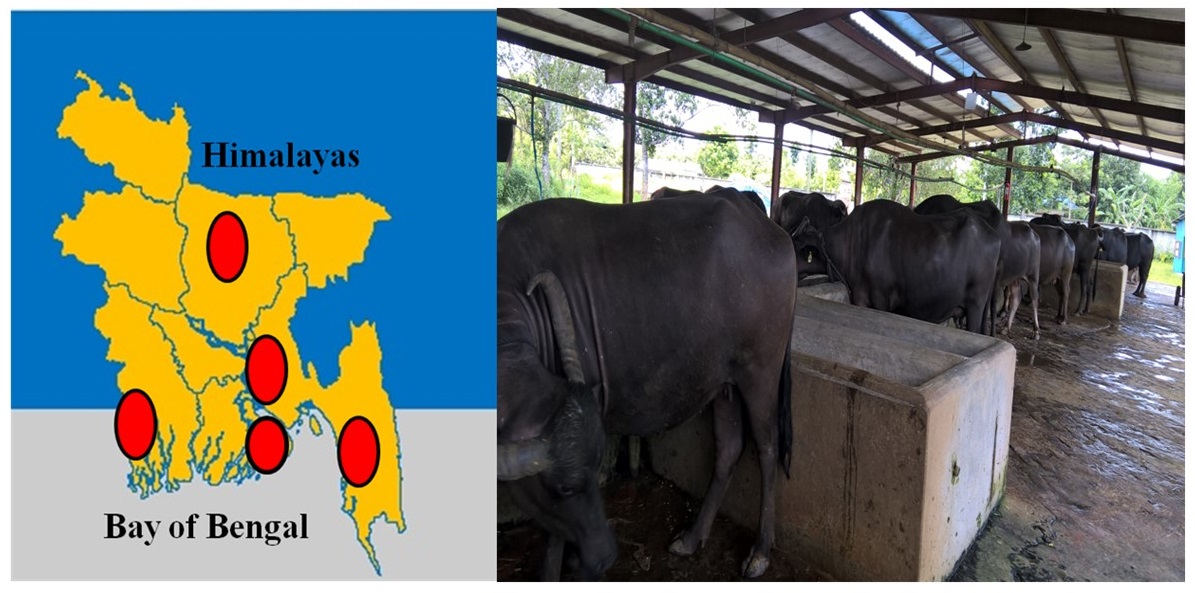

Supplement: Supplementary file 1 — Supplementary Information [file VMS3-8-2631-s001.jpg]

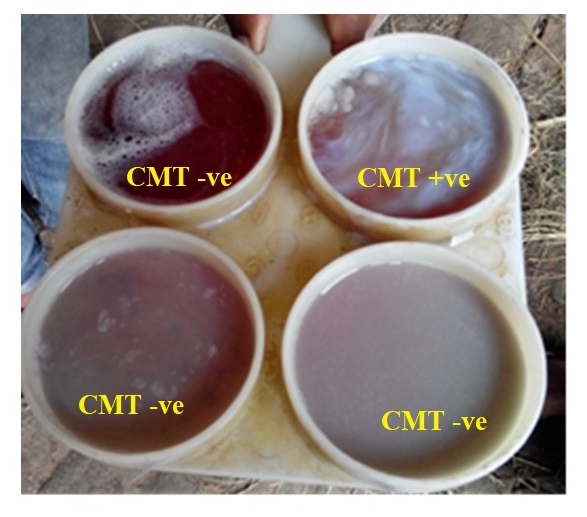

Supplement: Supplementary file 2 — Supplementary Information [file VMS3-8-2631-s002.jpg]
