# Supplementary material for: Antibiogram and virulence profiling reveals multidrug resistant Staphylococcus aureus as the predominant aetiology of subclinical mastitis in riverine buffaloes
Source: Vet Med Sci. 2022 Sep 22;8(6):2631–45. doi: 10.1002/vms3.942 (PMC9677375; doi:10.1002/vms3.942)
Supplement: Supplementary file 3 — Supplementary Information [file VMS3-8-2631-s003.docx]

| **Gene** | **Froward primer (5´-3´)** | **Reverse primer (3´-5´)** | **Product size (bp)** | **Primer set** |
| --- | --- | --- | --- | --- |
| *nuc* | GCGATTGATGGTGATACGGTT | AGCCAAGCCTTGACGAACTAAAGC | 279 | 1 |
| *sea* | GGTTATCAATGTGCGGGTGG | CGGCACTTTTTTCTCTTCGG | 102 | 2 |
| *seb* | GTATGGTGGTGTAACTGAGC | CCAAATAGTGACGAGTTAGG | 164 | 2 |
| *sec* | AGATGAAGTAGTTGATGTGTATGG | CACACTTTTAGAATCAACCG | 491 | 2 |
| *sed* | CCAATAATAGGAGAAAATAAAAG | ATTGGTATTTTTTTTCGTTC | 495 | 1 |
| *see* | AGGTTTTTTCACAGGTCATCC | CTTTTTTTTCTTCGGTCAATC | 430 | 1 |
| *mecA* | GTAGAAATGACTGAACGTCCGATAA | CCAATTCCACATTGTTTCGGTCTAA | 163 | 1 |
| *pvl* | ATCATTAGGTAAAATGTCTGGACATGATCCA | GCATCAAGTGTATTGGATAGCAAAAGC | 433 | 1 |
| *tsst-*1 | ACCCCTGTTCCCTTATCATC | TTTTCAGTATTTGTAACGCC | 326 | 1 |
| *eta* | ATATCAACGTGAGGGCTCTAGTAC | ATGCAGTCAGCTTCTTACTGCTA | 93 | 2 |
| *etb* | CACACATTACGGATAATGCAAG | TCAACCGAATAGAGTGAACTTATCT | 226 | 2 |

Table S1: Primer sequences, anticipated product size, and multiplex PCR sets used for identification of *Staphylococcus aureus* genes.

* Nucleotide primers were selected from previously published sequence ([Carfora et al., 2015](#_ENREF_1); [Hoque, Das, Rahman, Haider, & Islam, 2018](#_ENREF_2); [Peles et al., 2007](#_ENREF_3); [Wang et al., 2012](#_ENREF_4)).

Reference

Carfora, V., Caprioli, A., Marri, N., Sagrafoli, D., Boselli, C., Giacinti, G., . . . Battisti, A. (2015). Enterotoxin genes, enterotoxin production, and methicillin resistance in Staphylococcus aureus isolated from milk and dairy products in Central Italy. *International Dairy Journal, 42*, 12-15.

Hoque, M., Das, Z., Rahman, A., Haider, M., & Islam, M. (2018). Molecular characterization of Staphylococcus aureus strains in bovine mastitis milk in Bangladesh. *International journal of veterinary science and medicine, 6*(1), 53-60.

Peles, F., Wagner, M., Varga, L., Hein, I., Rieck, P., Gutser, K., . . . Béri, B. (2007). Characterization of Staphylococcus aureus strains isolated from bovine milk in Hungary. *International journal of food microbiology, 118*(2), 186-193.

Wang, X., Meng, J., Zhang, J., Zhou, T., Zhang, Y., Yang, B., . . . Xia, X. (2012). Characterization of Staphylococcus aureus isolated from powdered infant formula milk and infant rice cereal in China. *International journal of food microbiology, 153*(1-2), 142-147.
