# Supplementary material for: Antibiogram and virulence profiling reveals multidrug resistant Staphylococcus aureus as the predominant aetiology of subclinical mastitis in riverine buffaloes
Source: Vet Med Sci. 2022 Sep 22;8(6):2631–45. doi: 10.1002/vms3.942 (PMC9677375; doi:10.1002/vms3.942)
Supplement: Supplementary file 4 — Supplementary Information [file VMS3-8-2631-s004.docx]

**Fig. S1:** Selected study areas and buffalo farms.

**Fig. S2:** On farm screening test for subclinical mastitis diagnosis in buffalo cows.
